# Supplementary material for: SWI/SNF ATPase silenced HLF potentiates lung metastasis in solid cancers
Source: Nat Commun. 2025 Jun 5;16:5226. doi: 10.1038/s41467-025-60329-9 (PMC12141477; doi:10.1038/s41467-025-60329-9)
Supplement: Supplementary file 1 — Supplementary Information [file 41467_2025_60329_MOESM1_ESM.pdf]

Supplementary information

SWI/SNF ATPase silenced *HLF* potentiates lung metastasis in solid cancers

Jin Zhou[1], Austin Hepperla[2,3,4], Jeremy M. Simon[2,3,4], Kangsan Kim[1], Qing Hu[1], Chuanhai Zhang[5], Lei Dong[6], Lianxin Hu[1], Cheng Zhang[1], Chengheng Liao[1], Alice Fang[1], Yayoi Adachi[1], Haoyong Fu[1], Tao Wang[1], Qian Liang[1], Fangzhou Zhao[1], Hongyi Liu[1], Masashi Takeda[1], Jun Fang[1], Hua Zhong[1], Peter Ly[1], Lu Wang[7], Payal Kapur[1,8], Lin Xu[6], Liwei Jia[1], Srinivas Malladi[1], James Brugarolas[8,9], M. Celeste Simon[10 *], Bo Li[11 *], and Qing Zhang[1,8,12 *]

[1] Department of Pathology, University of Texas Southwestern Medical Center, Dallas, TX 75390, USA

[2] Lineberger Comprehensive Cancer Center, University of North Carolina School of Medicine, Chapel Hill, NC 27599, USA

[3] Department of Genetics, University of North Carolina, Chapel Hill, NC 27599, USA

[4] UNC Neuroscience Center, University of North Carolina, Chapel Hill, NC 27599, USA

[5] Department of Physiology, University of Texas Southwestern Medical Center, Dallas, TX 75390, USA

[6] Quantitative Biomedical Research Center, Department of Health Data Science and Biostatistics, Peter O'Donnell Jr. School of Public Health, University of Texas Southwestern Medical Center, Dallas, TX 75390, USA.

[7]Department of Biochemistry and Molecular Genetics, Feinberg School of Medicine, Northwestern University, Chicago, IL 60611, USA

[8] Kidney Cancer Program, Simmons Comprehensive Cancer Center, University of Texas Southwestern Medical Center, Dallas, TX 75390, USA

[9] Department of Internal Medicine, University of Texas Southwestern Medical Center, Dallas, TX 75390, USA

[10] Abramson Family Cancer Research Institute, Department of Cell and Developmental Biology, University of Pennsylvania, Philadelphia, PA 19104, USA

[11] Department of Biochemistry and Molecular Biology, Cancer Research Institute, School of Basic Medical Sciences, Southern Medical University, Guangzhou 510515, P. R. China

*Co-corresponding Authors: M. Celeste Simon (celeste2@pennmedicine.upenn.edu), Bo Li (libo47@smu.edu.cn) and Qing Zhang (Qing.Zhang@UTSouthwestern.edu)

[12] Lead contact

Supplementary Figures 1–10

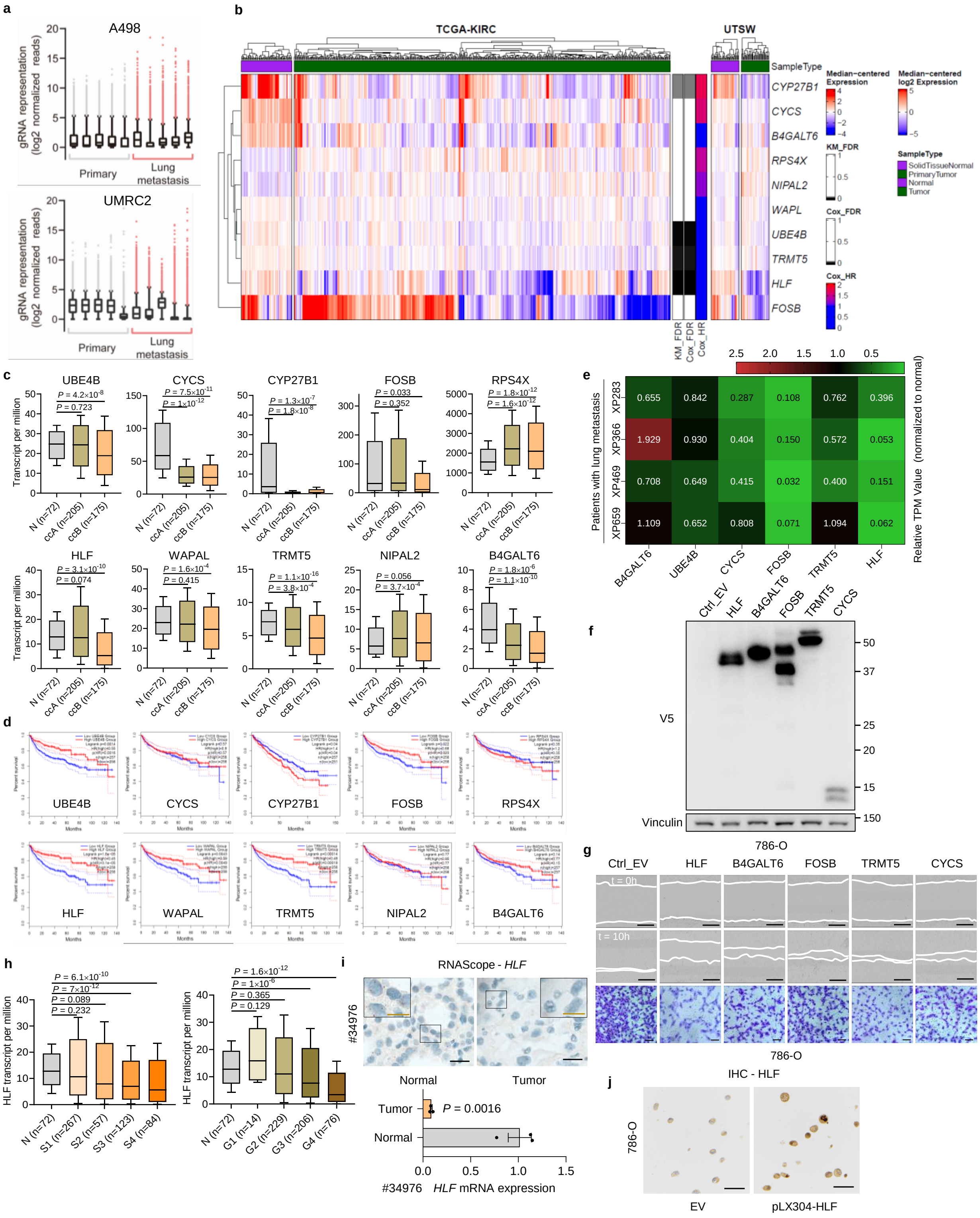

a
A498
gRNA representation (log2 normalized reads)
Primary
Lung metastasis
UMRC2
b
TCGA-KIRC
UTSW
SampleType
CYP27B1
CYCS
B4GALT6
RPS4X
NIPAL2
WAPL
UBE4B
TRMT5
HLF
FOSB
KM_FDR
Cox_FDR
Cox_HR
Median−centered Expression
Median−centered log2 Expression
SampleType
SolidTissueNormal
PrimaryTumor
Normal
Tumor
c
UBE4B
CYCS
CYP27B1
FOSB
RPS4X
HLF
WAPAL
TRMT5
NIPAL2
B4GALT6
Transcript per million
N (n=72)
ccA (n=205)
ccB (n=175)
d
UBE4B
CYCS
CYP27B1
FOSB
RPS4X
HLF
WAPAL
TRMT5
NIPAL2
B4GALT6
Percent survival
Months
e
Patients with lung metastasis
XP283
XP366
XP469
XP659
B4GALT6
UBE4B
CYCS
FOSB
TRMT5
HLF
Relative TPM Value (normalized to normal)
f
Ctrl_EV
HLF
B4GALT6
FOSB
TRMT5
CYCS
V5
Vinculin
g
786-O
Ctrl_EV
HLF
B4GALT6
FOSB
TRMT5
CYCS
t = 0h
t = 10h
786-O
h
HLF transcript per million
N (n=72)
S1 (n=267)
S2 (n=57)
S3 (n=123)
S4 (n=84)
G1 (n=14)
G2 (n=229)
G3 (n=206)
G4 (n=76)
i
RNAScope - HLF
#34976
Normal
Tumor
P = 0.0016
#34976 HLF mRNA expression
j
IHC - HLF
786-O
EV
pLX304-HLF

Supplementary Fig.1 Validation of potential metastatic suppressors identified from *in vivo* genome-wide CRISPR-Cas9 screening.

a Boxplot showing the sgRNA distribution of the human GeCKOv2 library in harvested tissue samples of primary tumors and lung metastatic tumors (n=5 biological replicates).

b Heatmap of the mRNA level of potential positive hits from TCGA database (normal, n=72; KIRC, n=533) and database from UTSW Kidney Cancer Specialized Program of Research Excellence (SPORE) program (n=41 pairs of normal and ccRCC tumor tissues).

c The mRNA level (transcript per million) of the indicated genes in KIRC patients based on ccRCC subtypes (ccA, n=205, ccB, n=175) compared with normal (N, n=72).

d Disease-free survival data of the indicated genes from the cancer genome atlas kidney renal clear cell carcinoma (TCGA-KIRC) dataset.

e RT-qPCR (n=3 biological replicates) quantification of the mRNA level of the indicated genes in normal and ccRCC tumor tissues (patients with lung metastasis), n=4 pairs of samples.

f, **g** Immunoblotting analysis (**f**), representative images of wound healing assay and transwell invasion assay (**g**) in 786-O cells transduced with pLX304-empty vector (Ctrl_EV) or target genes cloned into pLX304 backbone, n = 3 independent experiments. Wound healing, scale bar, 400 μm; transwell invasion, scale bar, 100 μm.

h The mRNA level of *HLF* in normal and KIRC tissues based on individual cancer stage (S) and tumor grade (G) in TCGA dataset, sample sizes were indicated in the figure.

i Representative images of *HLF* RNAScope in situ hybridization (ISH) and corresponding *HLF* mRNA expression in paired normal and ccRCC tumor tissues, used for validation of the specificity of the *HLF* probe, n= 3 biological replicates. Scale bar, 20 μm (10 μm for zoomed-in views).

j Representative images of HLF immunohistochemistry (IHC) in 786-O cell blocks overexpressed with empty vector (EV) or pLX304-V5-*HLF*, used for validation of the specificity of the HLF antibody. Scale bar, 50 μm.

Data are mean ± s.e.m. (i), box plots show the median and interquartile range, and whiskers show the data range (c, h), heatmap in e shows the relative data in tumors compared to normal tissues. Paired two-tailed t-test (i), exact *P* values are indicated. Representative immunoblots shown in figures were repeated at least two times independently with similar results. Source data are provided as a Source Data file.

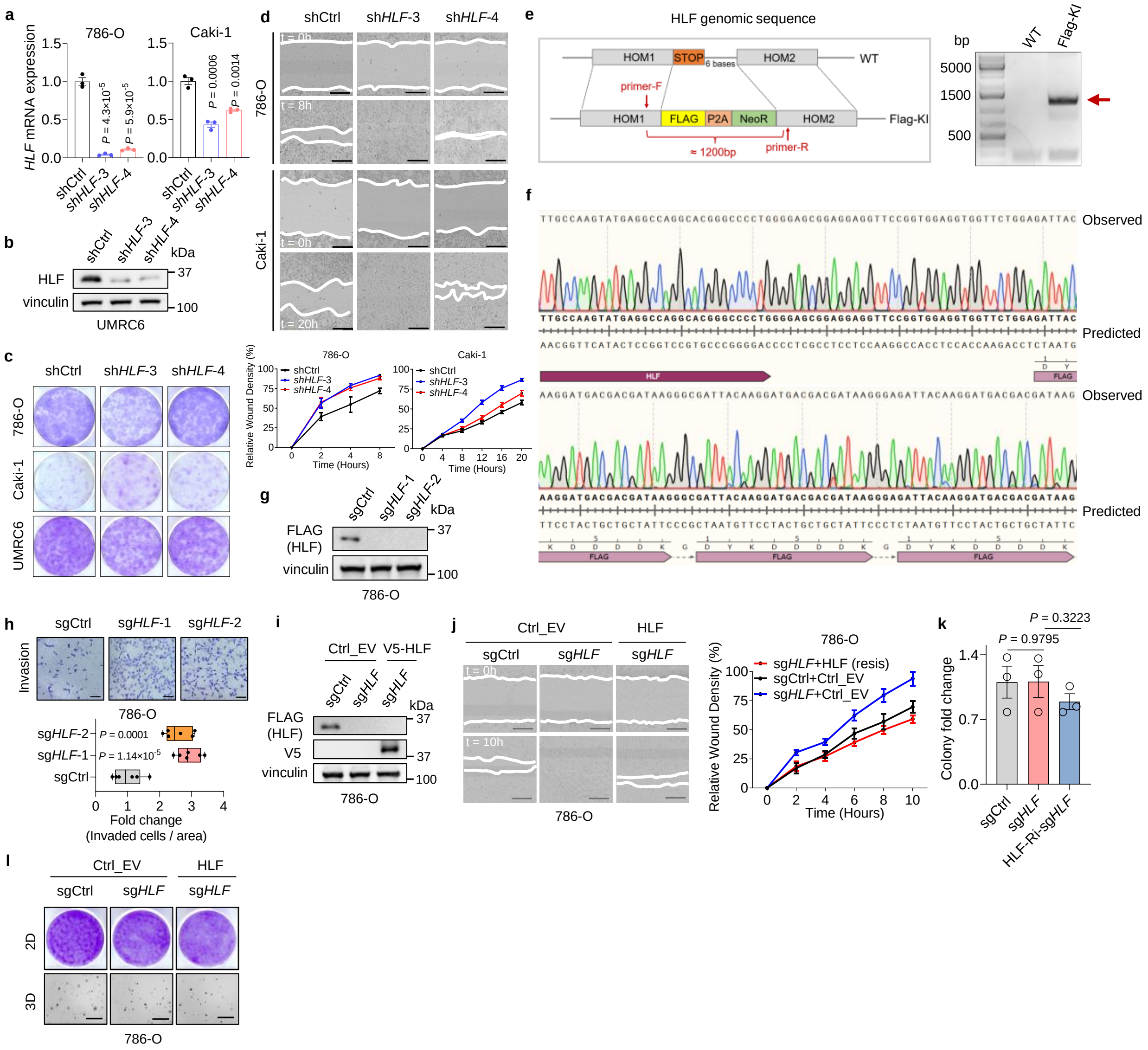

a
786-O
Caki-1
HLF mRNA expression
P = 4.3×10-5
P = 5.9×10-5
P = 0.0006
P = 0.0014
shCtrl
shHLF-3
shHLF-4
b
HLF
vinculin
kDa
UMRC6
c
786-O
Caki-1
UMRC6
d
shCtrl
shHLF-3
shHLF-4
t = 0h
t = 8h
t = 20h
Relative Wound Density (%)
Time (Hours)
e
HLF genomic sequence
HOM1
STOP
6 bases
HOM2
WT
primer-F
FLAG
P2A
NeoR
Flag-KI
≈ 1200bp
primer-R
bp
5000
1500
500
WT
Flag-KI
f
Observed
Predicted
HLF
FLAG
g
FLAG (HLF)
vinculin
sgCtrl
sgHLF-1
sgHLF-2
786-O
h
sgCtrl
sgHLF-1
sgHLF-2
Invasion
sgHLF-2
P = 0.0001
sgHLF-1
P = 1.14×10-5
Fold change (Invaded cells / area)
i
Ctrl_EV
V5-HLF
V5
j
Ctrl_EV
HLF
sgCtrl
sgHLF
t = 10h
sgHLF+HLF (resis)
sgCtrl+Ctrl_EV
sgHLF+Ctrl_EV
k
P = 0.3223
P = 0.9795
Colony fold change
HLF-Ri-sgHLF
l
2D
3D

Supplementary Fig.2 *HLF* depletion promotes ccRCC cells invasion without affecting cell proliferation.

a-d RT-qPCR quantification (n = 3 biological replicates) (**a**), immunoblotting analysis (**b**), 2D colony formation assay (**c**), representative images and quantification (**d**) of wound healing assay performed in the indicated cell lines transduced with shCtrl or *HLF* shRNAs (sh*HLF*-3/4). The samples derive from the same experiment but different gels were processed in parallel.

e Schematic of inserting a FLAG tag at the C terminus of HLF in the genome via knock-in (KI) in 786-O cells, along with PCR genotyping confirmation of the FLAG tag KI.

f Sanger sequencing confirmation of the FLAG tag knock-in in 786-O cells.

g, **h** Immunoblotting analysis (**g**), representative images and quantification (**h**) of transwell invasion assay in 786-O FLAG-KI cells transduced with sgCtrl or *HLF* sgRNAs (sg*HLF*-1/2), n=6 independent cell cultures. Scale bar, 100 μm.

i-l Immunoblotting (**i**), representative images and quantification (n=5 independent cell culture samples) (**j**) of wound healing assay, soft agar quantification (n=3 biological replicates) (**k**) and corresponding representative images of 2D colony formation and soft agar (**l**) in 786-O FLAG-KI cells overexpressed with empty vector or V5-sg*HLF*-resistant-*HLF* followed by infection with sgCtrl or sg*HLF*. Scale bar, wound healing, 400 μm; soft agar assay, 1mm. The samples derive from the same experiment but different gels for FLAG, Vinculin and another for V5 were processed in parallel.

Data are mean ± s.e.m. (a, d, j, k), box plots show the median and interquartile range, and whiskers show the data range (h). One-way ANOVA followed by a post hoc Dunnett-t-test (a, h, k), exact *P* values are indicated. Representative immunoblots shown in figures were repeated at least two times independently with similar results. Source data are provided as a Source Data file.

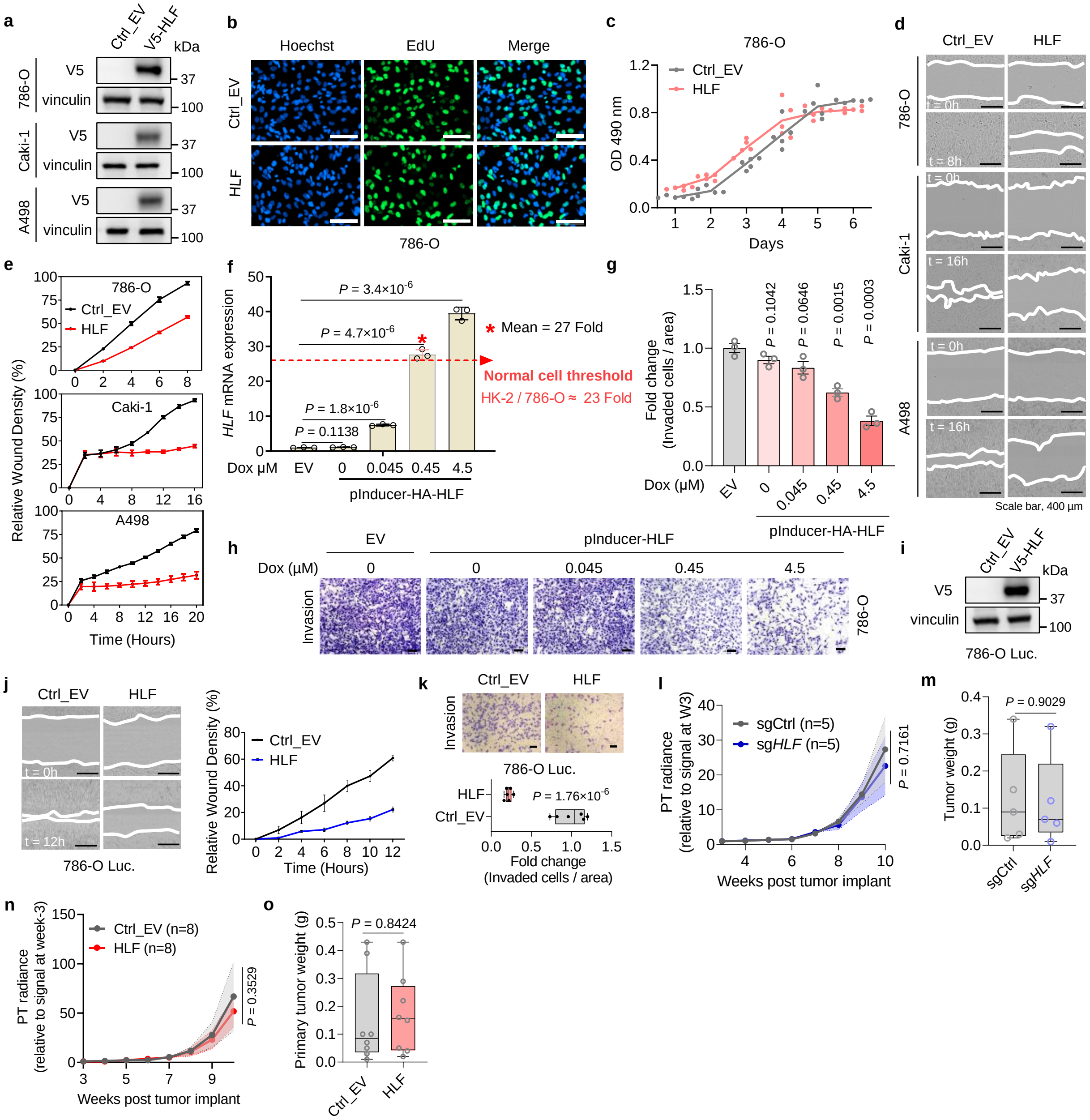

a
Ctrl_EV
V5-HLF
kDa
786-O
V5
vinculin
Caki-1
A498
37
100
b
Hoechst
EdU
Merge
Ctrl_EV
HLF
786-O
c
786-O
Ctrl_EV
HLF
OD 490 nm
Days
d
Ctrl_EV
HLF
t = 0h
t = 8h
t = 16h
Caki-1
A498
Scale bar, 400 µm
e
786-O
Caki-1
A498
Relative Wound Density (%)
Time (Hours)
f
HLF mRNA expression
P = 3.4×10^-6
P = 4.7×10^-6
P = 1.8×10^-6
P = 0.1138
* Mean = 27 Fold
Normal cell threshold
HK-2 / 786-O ≈ 23 Fold
Dox µM
EV
pInducer-HA-HLF
g
Fold change (Invaded cells / area)
P = 0.1042
P = 0.0646
P = 0.0015
P = 0.0003
Dox (µM)
h
Dox (µM)
pInducer-HLF
Invasion
i
786-O Luc.
j
t = 12h
k
Fold change (Invaded cells / area)
P = 1.76×10^-6
l
sgCtrl (n=5)
sgHLF (n=5)
PT radiance (relative to signal at W3)
Weeks post tumor implant
P = 0.7161
m
Tumor weight (g)
P = 0.9029
sgCtrl
sgHLF
n
Ctrl_EV (n=8)
HLF (n=8)
PT radiance (relative to signal at week-3)
P = 0.3529
o
Primary tumor weight (g)
P = 0.8424

Supplementary Fig.3 *HLF* overexpression inhibits ccRCC cells sheet migration and invasion without affecting cell proliferation.

a-e Immunoblotting analysis (**a**), EdU cell proliferation assay (**b**), MTS proliferation assay (**c**), representative images (**d**) and quantification (**e**) of wound healing assay, in the indicated cell lines overexpressed with empty vector (Ctrl_EV) or pLX304-V5-*HLF*. For EdU assay, cells treated with 10 μM EdU for 8 h were detected with Andy Fluor™ 488 azide (green) and counterstained with Hoechst (blue). n = 3 independent experiments. Scale bar, EdU,100 μm; wound healing, 400 μm.

f-h RT-qPCR quantification (**f**), quantification (**g**) and representative images (**h**) of transwell invasion assay in 786-O cells overexpressed with empty vector (Ctrl_EV) or pInducer-HA-*HLF* followed by doxycycline (Dox) treatment for 48h. The comparable overexpression level of *HLF* in 786-O cells to normal cells HK-2 is shown. n = 3 biological replicates. Scale bar, 200 μm.

i-k Immunoblotting analysis (**i**), representative images and quantification (**j**) of wound healing (n=5 independent cell cultures), representative images and quantification (**k**) of transwell invasion assay (n=6 independent cell cultures) in 786-O luciferase stable cells overexpressed with empty vector (Ctrl_EV) or pLX304-V5-*HLF*. Scale bar, wound healing, 400 μm; transwell invasion, 200 μm.

l, **m** Quantification of bioluminescence imaging of mice post signal stability (**l**) and tumor weight (**m**) in luciferase-labeled 786-O cells transduced with sgCtrl or sg*HLF* followed by orthotopic injection into the renal sub-capsule of NSG mice, n=5 mice in each group.

n, o Quantification of bioluminescence imaging of mice post signal stability (**n**) and primary tumor weight (**o**) after dissection of mice from 786-O luciferase stable cell lines that transduced with empty vector (EV) or pLX304-V5-*HLF* followed by orthotopic injection into the renal sub-capsule of NSG mice (n=8 mice in each group).

Data are mean ± s.e.m. (e, f, g, j, l, n), box plots show the median and interquartile range, and whiskers show the data range (k, m, o), XY graph in c shows all values. One-way ANOVA followed by a post hoc Dunnett-t-test (f, g) or unpaired two-tailed t-test (k, l-o), exact *P* values are indicated. Representative immunoblots shown in figures were repeated at least two times independently with similar results. Source data are provided as a Source Data file.

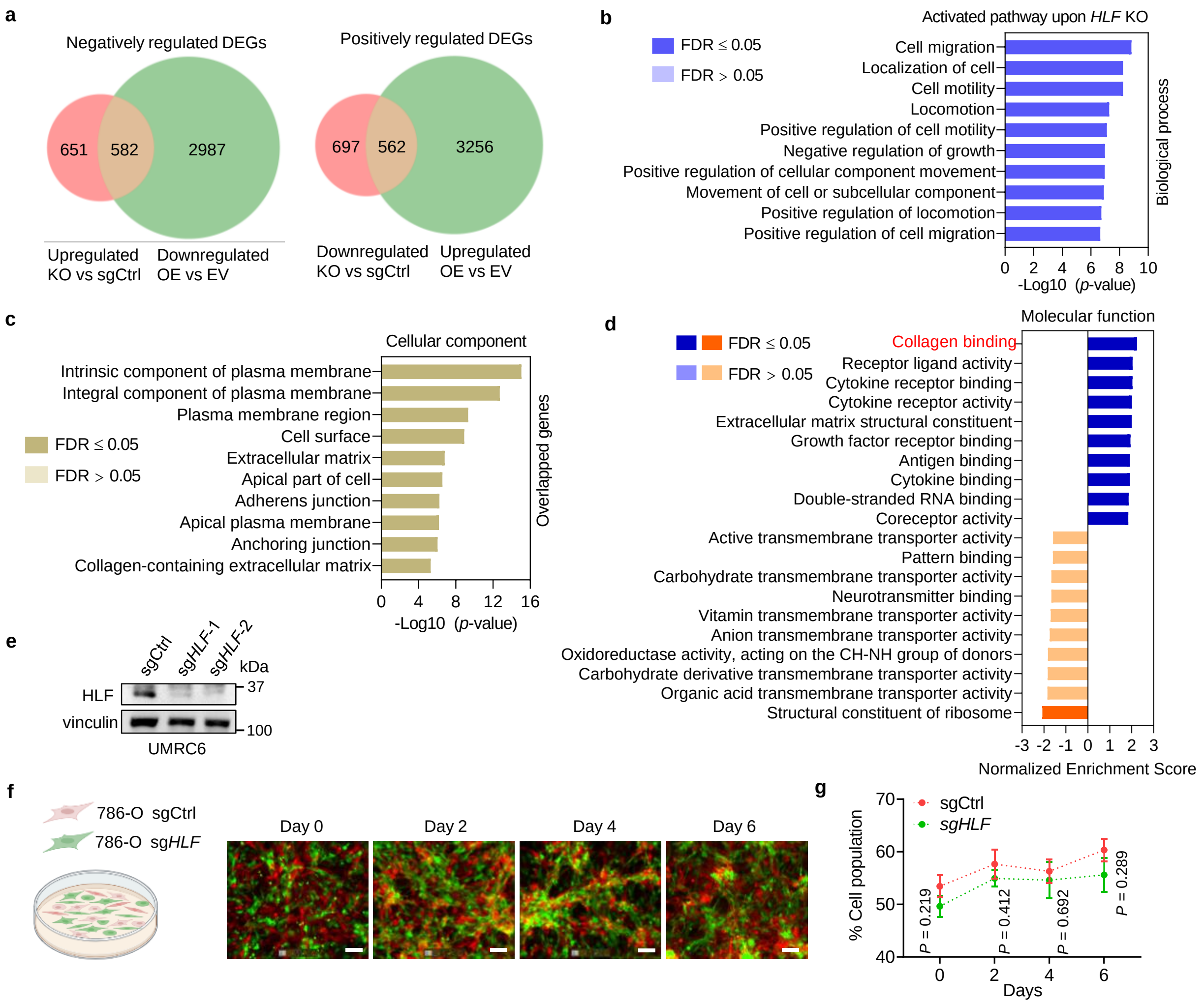

Supplementary Fig.4 Cell-collagen/matrix interaction mediates the inhibitory function of HLF in cell invasion capability.

a Venn diagram showing the overlapped DEGs between two batches of RNA-seq data (*HLF* overexpression group and knockout group) that are regulated by *HLF* consistently.

b GO analysis of negatively regulated genes by HLF from OE/KO RNA-seq data in terms of biological process.

c GO analysis of overlapped genes (258 genes) with Log2FC = ±0.5 in both *HLF* overexpression and knock-out group in terms of cellular component.

d GSEA analysis of the *HLF*-OE vs EV seq data in terms of molecular function.

e Immunoblotting of UMRC6 cells transduced with sgCtrl or *HLF* sgRNAs.

f, **g** Cell competition assay was performed in RFP-labeled sgCtrl cells and GFP-labeled sg*HLF* cells. Scheme of co-culture and fluorescence imaging (**f**) of live cells at indicated time points, and corresponding quantification (**g**) of cell population (n=6 for D0/D2, n=5 for D4 and n=3 for D6 from

independent cell cultures). Scale bar, 100 μm. Created in BioRender. *Zhou, J.* (2025) https://BioRender.com/x5scj3g.

Data are mean ± s.e.m. (g), unpaired two-tailed t-test (g), exact *P* values are indicated. Representative immunoblots shown in figures were repeated at least two times independently with similar results. Source data are provided as a Source Data file.

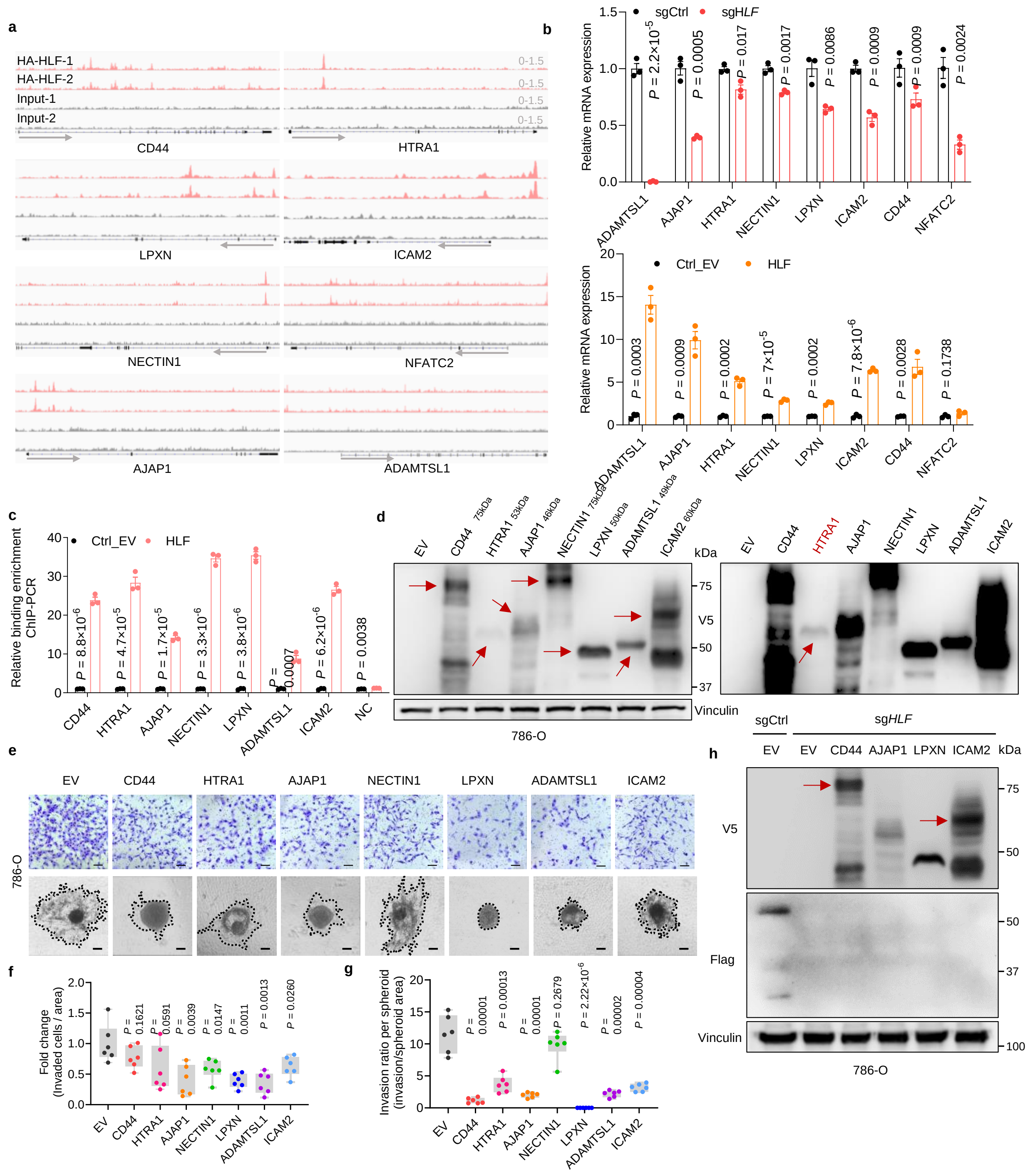

Supplementary Fig.5 Identification and validation of HLF downstream targets.

a ChIP-seq binding peaks of HA-tagged HLF on the indicated genes, n=2 replicates.

b RT-qPCR quantification of the HLF-targeted genes in 786-O cells transduced with empty vector (Ctrl_EV) / pLX304-V5-*HLF* or sgCtrl / sg*HLF*, n=3 biological replicates.

c ChIP-PCR validation of HA-HLF enrichment at the target genes compared to empty vector control (Ctrl_EV), an inactive intragenic region served as the negative control (NC), n=3 biological replicates.

d-**g** Immunoblotting analysis (**d**), representative images of transwell invasion assay and 3D spheroid invasion assay (**e**), quantification of transwell invasion assay (**f**) and quantification of 3D spheroid invasion (**g**) in 786-O cells transduced with pLX304-empty vector (Ctrl_EV) or HLF-targeted genes cloned into pLX304 backbone. Increased exposure was shown on the right to indicate the overexpression of HTRA1 protein. Spheroids were cultured in collagen for 3 days. n=6 independent cell cultures, scale bar, 100μm.

h Immunoblotting analysis in 786-O cells transduced with sgCtrl or sg*HLF*, followed by infection with lentivirus expressing pLX304-empty vector (EV) or HLF-targeted genes in pLX304 backbone. The samples derive from the same experiment but different gels for FLAG, Vinculin and another for V5 were processed in parallel.

Data are mean ± s.e.m. (b, c), box plots show the median and interquartile range, and whiskers show the data range (f, g). One-way ANOVA followed by a post hoc Dunnett-t-test (f, g) or unpaired two-tailed t-test (b, c), exact *P* values are indicated. Representative immunoblots shown in figures were repeated at least two times independently with similar results. Source data are provided as a Source Data file.

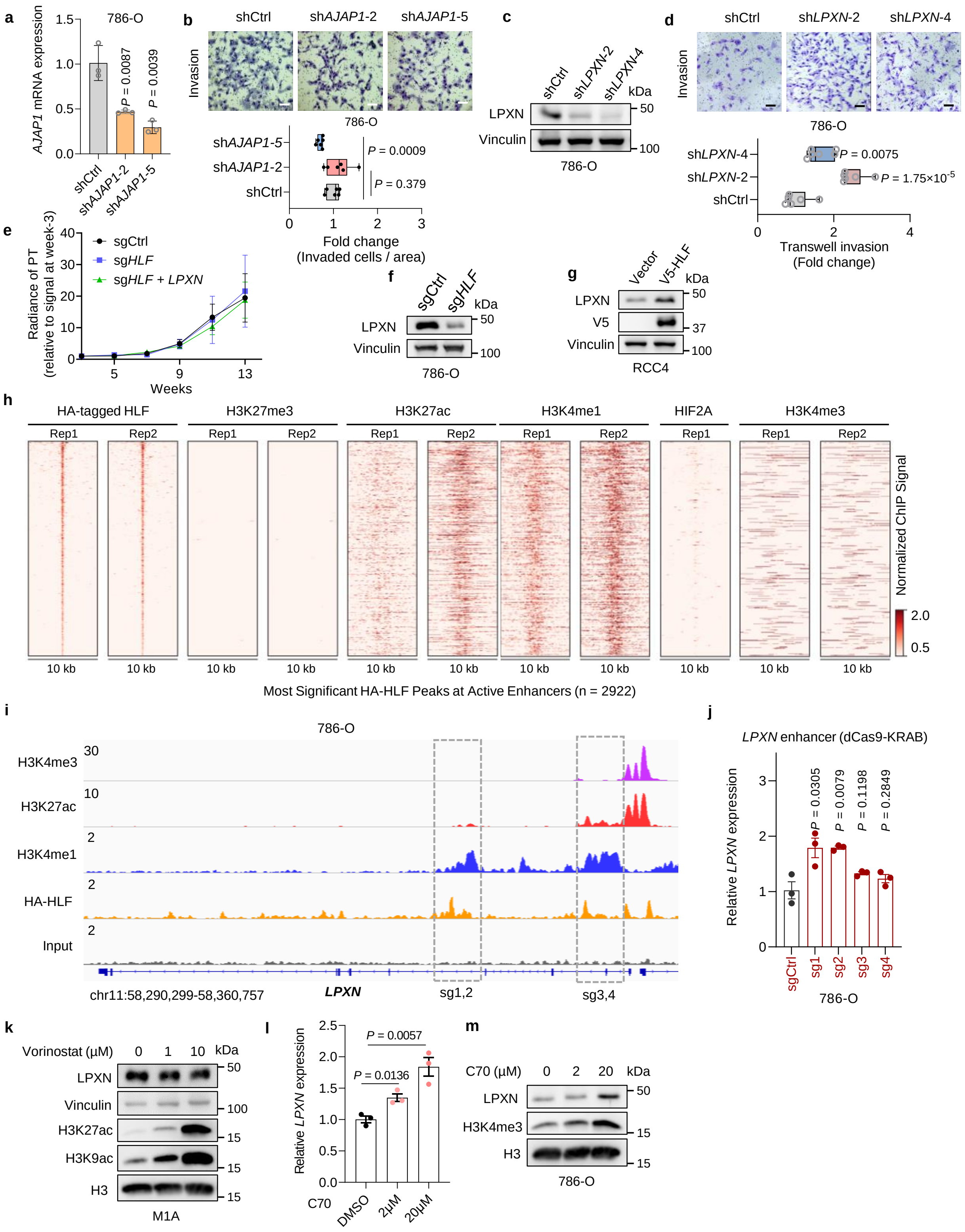

a
786-O
AJAP1 mRNA expression
P = 0.0087
P = 0.0039
shCtrl
shAJAP1-2
shAJAP1-5
b
shCtrl
shAJAP1-2
shAJAP1-5
Invasion
786-O
P = 0.0009
P = 0.379
Fold change
(Invaded cells / area)
c
shCtrl
shLPXN-2
shLPXN-4
kDa
LPXN
Vinculin
786-O
d
shCtrl
shLPXN-2
shLPXN-4
Invasion
786-O
P = 0.0075
P = 1.75×10-5
Transwell invasion
(Fold change)
e
sgCtrl
sgHLF
sgHLF + LPXN
Radiance of PT
(relative to signal at week-3)
Weeks
f
sgCtrl
sgHLF
LPXN
Vinculin
786-O
g
Vector
V5-HLF
LPXN
V5
Vinculin
RCC4
h
HA-tagged HLF
H3K27me3
H3K27ac
H3K4me1
HIF2A
H3K4me3
Rep1
Rep2
Normalized ChIP Signal
10 kb
Most Significant HA-HLF Peaks at Active Enhancers (n = 2922)
i
786-O
H3K4me3
H3K27ac
H3K4me1
HA-HLF
Input
chr11:58,290,299-58,360,757
LPXN
sg1,2
sg3,4
j
LPXN enhancer (dCas9-KRAB)
Relative LPXN expression
P = 0.0305
P = 0.0079
P = 0.1198
P = 0.2849
sgCtrl
sg1
sg2
sg3
sg4
786-O
k
Vorinostat (μM)
LPXN
Vinculin
H3K27ac
H3K9ac
H3
M1A
l
Relative LPXN expression
P = 0.0057
P = 0.0136
C70
DMSO
2μM
20μM
m
C70 (μM)
LPXN
H3K4me3
H3
786-O

Supplementary Fig.6 Validation of LPXN as the critical downstream target of HLF.

a, **b** RT-qPCR quantification (n = 3 biological replicates) (**a**), representative images and quantification (**b**) of transwell invasion (n=6 independent cell cultures) in 786-O cells transduced with shCtrl or *AJAP1* shRNAs (sh*AJAP1*-2/5). Scale bar, 100μm.

c, **d** Immunoblotting (**c**), representative images and quantification of transwell invasion assay (**d**) in 786-O cells transduced with shCtrl or sh*LPXN*-1/2, n=6 independent cell culture samples. Scale bar, 100μm.

e Quantification of bioluminescence imaging of mice post signal stability from luciferase-labeled 786-O cells transduced with sgCtrl or sg*HLF* followed by infection with lentivirus expressing pLX304-empty vector or pLX304-*LPXN*, and orthotopic injection into the renal sub-capsule of NSG mice, n=5 mice in each group.

f, **g** Immunoblotting analysis of 786-O cells transduced with sgCtrl / sg*HLF* (**f**) or RCC4 cells transduced with lentivirus expressing empty vector or pLX304-V5-*HLF* (**g**). The samples derive from the same experiment but different gels for LPXN, Vinculin and another for V5 were processed in parallel.

h Integrated analyses of HA-tagged HLF ChIP-seq and ChIP-seqs including H3K27me3, H3K27ac, H3K4me1, HIF2α, and H3K4me3.

i ChIP-seq binding peaks of H3K4me3, H3K27ac, H3K4me1, and HA-tagged HLF in 786-O cells at chr11:58,290,299-58,360,757 (hg19). The targeting sites of sg*LPXN*s at the potential enhancer region are indicated by gray dotted rectangle.

j RT-qPCR quantification of *LPXN* mRNA level in 786-O cells transduced with sgCtrl or sg*LPXN*s targeting specific enhancer region in CRISPRi system (dCas9-KRAB), n=3 biological replicates.

k Immunoblotting in M1A cells after treatment with vorinostat for 12h at 0/1/10 μM. The samples derive from the same experiment but different gels for LPXN, Vinculin, another for H3K27ac, another for H3K9ac and another for H3 were processed in parallel.

l, **m** RT-qPCR quantification (**l**) and immunoblotting analysis (**m**) of 786-O cells after treatment with C70 for 12h at 0/2/20 μM, n=3 biological replicates. The samples derive from the same experiment but different gels for each target protein were processed in parallel.

Data are mean ± s.e.m. (a, e, j, l), box plots show the median and interquartile range, and whiskers show the data range (b, d). One-way ANOVA followed by a post hoc Dunnett-t-test (a, b, d, j, l), exact *P* values are indicated. Representative immunoblots shown in figures were repeated at least two times independently with similar results. Source data are provided as a Source Data file.

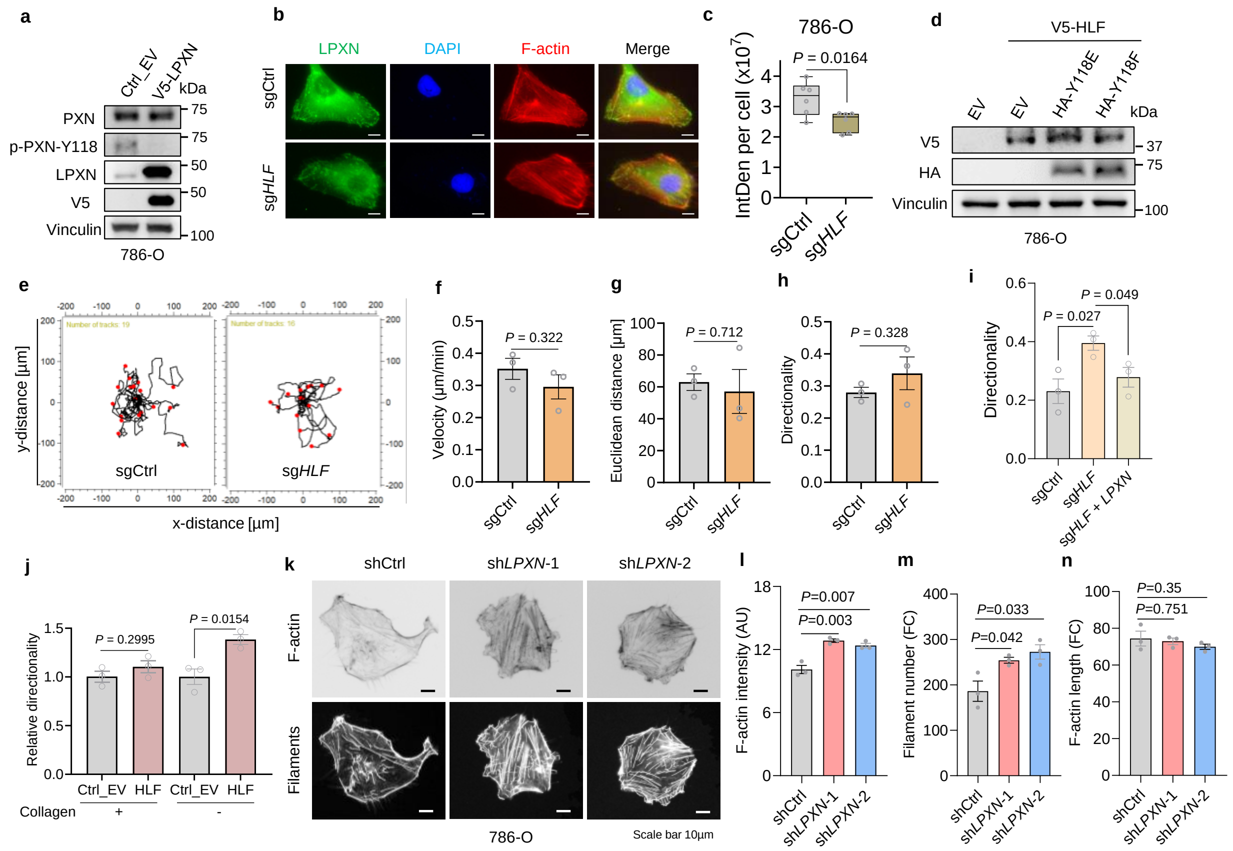

Supplementary Fig.7 The negative regulation of HLF in cell migration relies on the coordination of stiff collagen and actin cytoskeleton via LPXN-PXN.

a Immunoblotting analysis of 786-O cells overexpressed with empty vector (Ctrl_EV) or pLX304-V5-*LPXN*. The samples derive from the same experiment but different gels for PXN, LPXN, another for p-PXN-Tyr118, vinculin and another for V5 were processed in parallel.

b, **c** Immunofluorescence staining (**b**) and corresponding LPXN quantification (**c**) of 786-O cells transduced with sgCtrl or sg*HLF*. N=6 independent cell cultures. Scale bar, 10 µm.

d Immunoblotting analysis of 786-O cells overexpressed with empty vector (EV) or V5-pLX304-*HLF* followed by infection with Paxillin Y118E (phosphomimetic version) or Y118F (non-phosphorylatable version). The samples derive from the same experiment but different gels for HA, vinculin and another for V5 were processed in parallel.

e-h Wind-rose plots showing cell tracks (**e**), migration velocity (**f**), Euclidean distance (**g**) and directionality (**h**) of the tracked 786-O cells transduced with sgCtrl or sg*HLF* and cultured on surface without collagen coating. Cells from three independent cell cultures were analyzed.

i Directionality of the tracked 786-O cells transduced with sgCtrl or sg*HLF* followed by infection with lentivirus expressing pLX304-empty vector or pLX304-*LPXN* and cultured on stiff collagen surface, cells from three independent cell cultures were analyzed.

j Directionality of the tracked 786-O cells overexpressed with empty vector (Ctrl_EV) or pLX304-V5-*HLF* cultured on surface with or without stiff collagen coating (50 μg/ml collagen), the analyzed cells were from three independent cell cultures.

k-**n** Representative fluorescence images of F-actin and filaments (**k**), intensity of F-actin (**l**), filament number per cell (**m**), and length of F-actin (**n**) in 786-O cells transduced with shCtrl or sh*LPXN*-1/2 and cultured on stiff collagen surface (50 μg/ml collagen coating). Cells from three independent cell cultures were analyzed.

Data are mean ± s.e.m. (f-j, l-n), box plots show the median and interquartile range (c). One-way ANOVA followed by a post hoc Dunnett-t-test (i, l-n) or unpaired two-tailed t-test (f-h, j), exact *P* values are indicated. Representative immunoblots shown in figures were repeated at least two times independently with similar results. Source data are provided as a Source Data file.

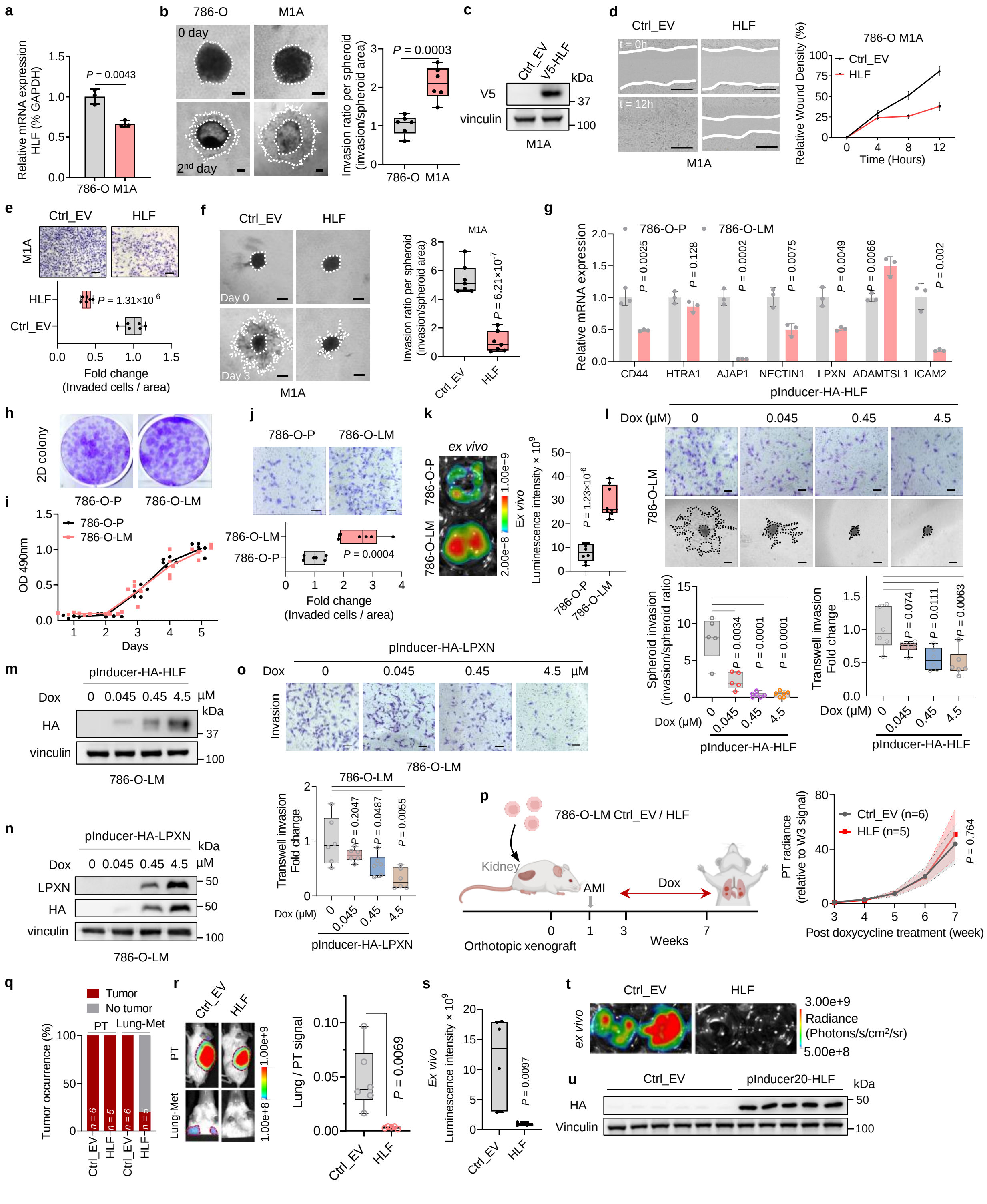

a
Relative mRNA expression HLF (% GAPDH)
P = 0.0043
786-O M1A
b
786-O
M1A
0 day
2nd day
Invasion ratio per spheroid (invasion/spheroid area)
P = 0.0003
c
Ctrl_EV
V5-HLF
kDa
V5
vinculin
37
100
M1A
d
Ctrl_EV
HLF
t = 0h
t = 12h
M1A
786-O M1A
Relative Wound Density (%)
Time (Hours)
e
M1A
P = 1.31×10^-6
Fold change (Invaded cells / area)
f
Day 0
Day 3
P = 6.21×10^-7
g
786-O-P
786-O-LM
Relative mRNA expression
CD44
HTRA1
AJAP1
NECTIN1
LPXN
ADAMTSL1
ICAM2
P = 0.0025
P = 0.128
P = 0.0002
P = 0.0075
P = 0.0049
P = 0.0066
P = 0.002
h
2D colony
786-O-P
786-O-LM
i
OD 490nm
Days
j
P = 0.0004
Fold change (Invaded cells / area)
k
ex vivo
Ex vivo Luminescence intensity × 10^9
P = 1.23×10^-6
l
pInducer-HA-HLF
Dox (μM)
0
0.045
0.45
4.5
Spheroid invasion (invasion/spheroid ratio)
P = 0.0034
P = 0.0001
P = 0.0001
Transwell invasion Fold change
P = 0.074
P = 0.0111
P = 0.0063
m
pInducer-HA-HLF
HA
vinculin
786-O-LM
n
pInducer-HA-LPXN
LPXN
HA
vinculin
786-O-LM
o
Invasion
786-O-LM
P = 0.2047
P = 0.0487
P = 0.0055
p
786-O-LM Ctrl_EV / HLF
Kidney
AMI
Dox
Weeks
Orthotopic xenograft
PT radiance (relative to W3 signal)
Ctrl_EV (n=6)
HLF (n=5)
P = 0.764
Post doxycycline treatment (week)
q
Tumor
No tumor
PT
Lung-Met
Tumor occurrence (%)
r
PT
Lung-Met
Lung / PT signal
P = 0.0069
s
Ex vivo Luminescence intensity × 10^9
P = 0.0097
t
ex vivo
Radiance (Photons/s/cm²/sr)
3.00e+9
5.00e+8
u
Ctrl_EV
pInducer20-HLF
HA
Vinculin

Supplementary Fig.8 *HLF* and *LPXN* overexpression inhibit cell invasion capability and lung metastasis in metastatic cell colonies.

a, **b** RT-qPCR (n=3 biological replicates) quantification of *HLF* mRNA level (**a**), representative images and corresponding quantification (**b**) of 3D spheroid invasion assay in 786-O and M1A cells, n=6 independent cell cultures. Scale bar, 100 μm.

c-**f** Immunoblotting (**c**), representative images and corresponding quantification (n=5 independent cell cultures) (**d**) of wound healing assay, representative images and quantification (n=6 independent cell cultures) (**e**) of transwell invasion assay, and representative images and quantification (n=6 independent cell cultures) (**f**) of 3D spheroid invasion assay in M1A cells overexpressed with empty vector (Ctrl_EV) or pLX304-V5-*HLF*. Scale bar, wound healing, 400 μm; transwell invasion and 3D collagen invasion, 200 μm.

g RT-qPCR quantification of 786-O-P and 786-O-LM cells, n=3 biological replicates.

h-**j** 2D colony formation assay (**h**), MTS proliferation assay (n=5 technical replicates) (**i**), representative images, and corresponding quantification (n=6 independent cell cultures) (**j**) of transwell invasion assay in 786-O-P and 786-O-LM cells. Scale bar, 100μm.

k Representative lung *ex vivo* images and quantification from luciferase-labeled 786-O-P, and 786-O-LM cells post tail vein inoculation into NSG mice, n=8 mice in each group.

l, **m** Representative images and quantification of transwell invasion and 3D collagen invasion (**l**), and immunoblotting analysis (**m**) in 786-O-LM cells overexpressed with pInducer-HA-*HLF* followed by doxycycline (Dox) treatment. N=6 independent cell cultures in each group. Scale bar, transwell invasion, 100μm, collagen invasion, 200μm. The samples derive from the same experiment but different gels were processed in parallel.

n, **o** Immunoblotting analysis (**n**), representative images and quantification of transwell invasion (**o**) in 786-O-LM cells overexpressed with pInducer-HA-*LPXN* followed by doxycycline (Dox) treatment for 48h, n=6 independent cell cultures. Scale bar, 100μm. The samples derive from the same experiment but different gels for HA, vinculin and another for LPXN were processed in parallel.

p-**u** Schematic and quantification of bioluminescence imaging of mice post signal stability (**p**), percentage of primary tumors (PT) and metastatic tumors (Lung-met) forming calculation (**q**), representative images of PT / Lung-met and signals of Lung-Met relative to its matching PT (**r**), quantification of lung *ex vivo* imaging (**s**) and corresponding representative images (**t**), immunoblotting of tumor samples after dissection of mice (**u**) from luciferase-labeled 786-O-LM cells overexpressed with empty vector (Ctrl_EV) or pInducer-HA-*HLF* followed by orthotopic injection into the renal sub-capsule of NSG mice (n=6 mice for Ctrl_EV and n=5 mice for *HLF* group), and doxycycline (dox) chow was treated with mice after week-3 when the luciferase signal was stable. Created in BioRender. *Zhou, J.* (2025) https://BioRender.com/x5scj3g.

Data are mean ± s.e.m. (a, d, g, p), box plots show the median and interquartile range, and whiskers show the data range (b, e, f, j, k, l, o, r, s), XY graph in **i** shows all values. One-way ANOVA followed by a post hoc Dunnett-t-test (l, o) or unpaired two-tailed t-test (a, b, e-g, j, k, p, r, s), exact *P* values are indicated. Representative immunoblots shown in figures were repeated at least two times independently with similar results. Source data are provided as a Source Data file.

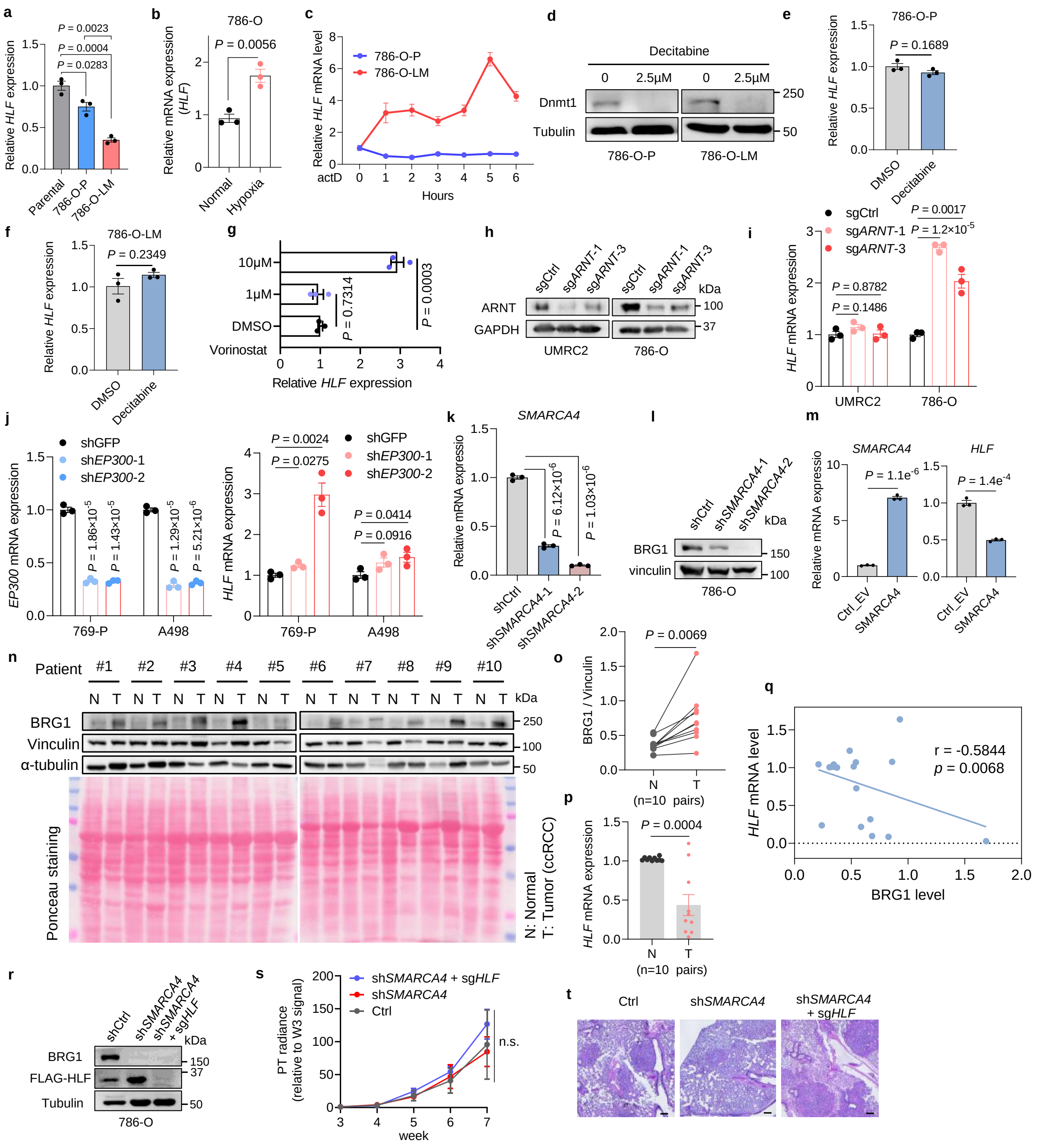
a
b
786-O
c
786-O-P
786-O-LM
Hours
d
Decitabine
Dnmt1
Tubulin
786-O-P
786-O-LM
e
786-O-P
P = 0.1689
f
786-O-LM
P = 0.2349
g
Vorinostat
Relative HLF expression
h
ARNT
GAPDH
UMRC2
786-O
i
sgCtrl
sgARNT-1
sgARNT-3
j
shGFP
shEP300-1
shEP300-2
769-P
A498
k
SMARCA4
l
BRG1
vinculin
m
SMARCA4
HLF
n
Patient
BRG1
Vinculin
α-tubulin
Ponceau staining
N: Normal
T: Tumor (ccRCC)
o
BRG1 / Vinculin
P = 0.0069
p
HLF mRNA expression
P = 0.0004
q
HLF mRNA level
BRG1 level
r = -0.5844
p = 0.0068
r
BRG1
FLAG-HLF
Tubulin
786-O
s
shSMARCA4 + sgHLF
shSMARCA4
Ctrl
PT radiance
(relative to W3 signal)
week
n.s.
t
Ctrl
shSMARCA4
shSMARCA4 + sgHLF

Supplementary Fig.9 Validation of the factors contributing to *HLF* loss.

a RT-qPCR quantification of *HLF* mRNA level in parental 786-O cells before orthotopic injection, 786-O-P and 786-O-LM cells, n=3 biological replicates.

b RT-qPCR quantification of *HLF* mRNA level in 786-O cells treated with normal oxygen concentration (~20%) or hypoxia condition (1%) for 24h, n=3 biological replicates.

c RT-qPCR quantification of *HLF* mRNA level in mRNA stability assay after actinomycin D (actD) treatment (5 µg/ml) for indicated hours in 786-O-P and 786-O-LM cells, n=3 biological replicates.

d-f Immunoblotting (**d**) and RT-qPCR quantification (**e, f**) in 786-O-P and 786-O-LM cells treated with 2.5 µM decitabine for 24h, n=3 biological replicates.

g RT-qPCR quantification in M1A cells after treatment with vorinostat for 12h at 0/1/10 µM, n=3 biological replicates.

h, **i** Immunoblotting (**h**) and RT-qPCR quantification of *HLF* mRNA level (**i**) in UMRC2 and 786-O cells transduced with sgCtrl or *ARNT* sgRNAs, n=3 biological replicates.

j RT-qPCR quantification of *HLF* mRNA level in 769-P and A498 cells transduced with shCtrl or *EP300* shRNAs, n=3 biological replicates.

k, **l** RT-qPCR quantification (**k**) and immunoblotting (**l**) in 786-O cells transduced with shCtrl or *SMARCA4* shRNAs, n=3 biological replicates. The samples derive from the same experiment but different gels were processed in parallel.

m RT-qPCR quantification of *SMARCA4* and *HLF* mRNA level in UMRC6 cells overexpressed with empty vector (Ctrl_EV) or pLVX-*SMARCA4*, n=3 biological replicates.

n-q Immunoblotting analysis (**n**) and corresponding quantification (n=10 pairs of samples) (**o**), RT-qPCR analysis (n=3 biological replicates) (**p**) and Pearson's correlation analysis (**q**) in paired normal (N) and tumor (T) patients' samples.

r-t Immunoblotting analysis (**r**), quantification of bioluminescence imaging of mice post signal stability of primary tumors (PT) (**s**) and H&E staining of lung metastatic tumors (Lung-mets) (**t**) from luciferase-labeled 786-O cells transduced with shCtrl or sh*SMARCA4* followed by infection with lentivirus expressing sgCtrl or sg*HLF* and orthotopic injection into the renal sub-capsule of NSG mice, n=6 mice in Ctrl and sh*SMARCA4* group, n=5 mice in rescue group. Scale bar, 200 µm. The samples derive from the same experiment but different gels for BRG1, Tubulin and another for FLAG were processed in parallel.

Data are mean ± s.e.m., One-way ANOVA followed by a post hoc Dunnett-t-test (a, g, i, j, k, s) or unpaired two-tailed t-test (b, e, m) or paired two-tailed t-test (o, p), exact *P* values are indicated. Representative immunoblots shown in figures were repeated at least two times independently with similar results. Source data are provided as a Source Data file.

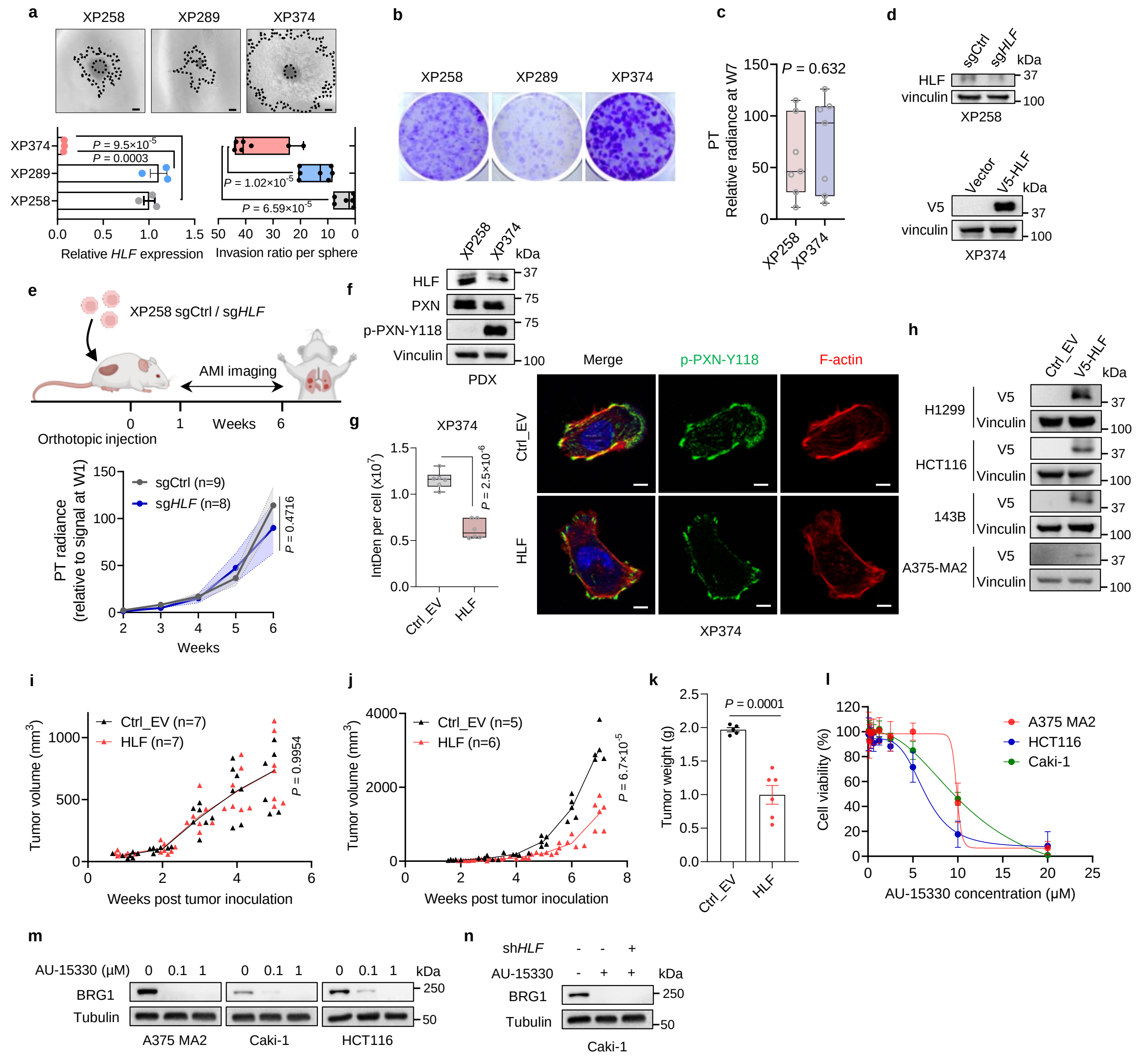

Supplementary Fig.10 HLF suppresses metastasis across multiple cancer types.

a RT-qPCR (n=3 biological replicates) quantification of *HLF* mRNA level, representative images, and quantification of 3D spheroid invasion assay in the indicated PDX cell lines after 3 days of culturing in collagen, n=6 independent cell cultures. Scale bar, 200μm.

b 2D colony formation assay of the indicated PDX cell lines.

c Quantification of bioluminescence imaging of primary tumor from luciferase-labeled XP258 and XP374 cells after orthotopic injection into NSG mice, n=7 mice in each group. The signal at the final week (W7), relative to week 3 when the signal stabilizes, was used to measure the tumor growth rate.

d Immunoblotting in XP258 cells transduced with sgCtrl / sg*HLF* or XP374 cells overexpressed with empty vector / pLX304-V5-*HLF*. The samples derive from the same experiment but different gels were processed in parallel.

e Schematic of orthotopic injection and quantification of bioluminescence imaging of mice post signal stability from luciferase-labeled XP258 cells that transduced with sgCtrl or sg*HLF* followed by orthotopic injection into NSG mice, n=9 mice in sgCtrl, n=8 mice in sg*HLF* group. Created in BioRender. *Zhou, J*. (2025) https://BioRender.com/x5scj3g.

f Immunoblotting of cell lysates from XP258 and XP374 cells. The samples derive from the same experiment but different gels for HLF, PXN vinculin and another for p-PXN-Y118 were processed in parallel.

g Representative fluorescence images of p-PXN-Y118 (green) and F-actin (red), and quantification of p-PXN-Y118 immunofluorescence in XP374 cells transduced with empty vector (EV) and pLX304-V5-*HLF* cultured on stiff surface (50 μg/ml collagen coating). Nucleus was stained with Hoechst (blue). Scale bar 5 μm. N=6 independent cell cultures.

h Immunoblotting analysis in the indicated cancer cell lines overexpressed with empty vector (Ctrl_EV) or pLX304-V5-*HLF* (V5-HLF). For H1299 cells, the samples derive from the same experiment but different gels were processed in parallel.

i Quantification of tumor size from HCT116 cells overexpressed with empty vector (Ctrl_EV) or pLX304-V5-*HLF* (HLF) followed by subcutaneous implantation in NSG mice, n=7 mice per group.

j, k Quantification of tumor volume (**j**) and tumor weight (**k**) from H1299 cells overexpressed with empty vector (Ctrl_EV) or pLX304-V5-*HLF* (HLF) followed by subcutaneous implantation in NSG mice, n=5 mice in Ctrl_EV group, n=6 mice in HLF group.

l Dose-response curves of A375 MA2, HCT116 and Caki-1 cells treated with AU-15330, n=5 technical replicates.

m Immunoblotting analysis in the indicated cell lines treated with AU-15330 (DMSO/0.1/1μM) for 24~48h.

n Immunoblotting analysis in Caki-1 cells transduced with shCtrl or sh*HLF*, followed by treatment with DMSO or 1μM AU-15330 for 48h.

Data are mean ± s.e.m. (a_left, e, k, l), box plots show the median and interquartile range, and whiskers show the data range (a_right, c, g), XY graph in i and j show all values. One-way ANOVA followed by a post hoc Dunnett-t-test (a) or unpaired two-tailed t-test (c, e, g, i-k), exact *P* values are indicated. Representative immunoblots shown in figures were repeated at least two times independently with similar results. Source data are provided as a Source Data file.
