# Supplementary material for: SWI/SNF ATPase silenced HLF potentiates lung metastasis in solid cancers
Source: Nat Commun. 2025 Jun 5;16:5226. doi: 10.1038/s41467-025-60329-9 (PMC12141477; doi:10.1038/s41467-025-60329-9)
Supplement: Supplementary file 2 — Description of Additional Supplementary Information [file 41467_2025_60329_MOESM2_ESM.pdf]

Description of Additional Supplementary Files

File Name: Supplementary Data 1
Description: Genes identified by both HLF overexpression (OE) and knockout (KO) RNAseq

File Name: Supplementary Data 2
Description: Oligonucleotide sequences
