# Supplementary material for: SWI/SNF ATPase silenced HLF potentiates lung metastasis in solid cancers
Source: Nat Commun. 2025 Jun 5;16:5226. doi: 10.1038/s41467-025-60329-9 (PMC12141477; doi:10.1038/s41467-025-60329-9)
Supplement: Supplementary file 5 — Reporting Summary [file 41467_2025_60329_MOESM5_ESM.pdf]

nature portfolio

Corresponding author(s): M. Celeste Simon, PhD
Bo Li, PhD
Qing Zhang, PhD

Last updated by author(s): May 7, 2025

Reporting Summary

Nature Portfolio wishes to improve the reproducibility of the work that we publish. This form provides structure for consistency and transparency in reporting. For further information on Nature Portfolio policies, see our Editorial Policies and the Editorial Policy Checklist.

Statistics

For all statistical analyses, confirm that the following items are present in the figure legend, table legend, main text, or Methods section.

n/a	Confirmed	
☐	☒	The exact sample size (*n*) for each experimental group/condition, given as a discrete number and unit of measurement
☐	☒	A statement on whether measurements were taken from distinct samples or whether the same sample was measured repeatedly
☐	☒	The statistical test(s) used AND whether they are one- or two-sided<br>*Only common tests should be described solely by name; describe more complex techniques in the Methods section.*
☒	☐	A description of all covariates tested
☐	☒	A description of any assumptions or corrections, such as tests of normality and adjustment for multiple comparisons
☐	☒	A full description of the statistical parameters including central tendency (e.g. means) or other basic estimates (e.g. regression coefficient) AND variation (e.g. standard deviation) or associated estimates of uncertainty (e.g. confidence intervals)
☒	☐	For null hypothesis testing, the test statistic (e.g. *F*, *t*, *r*) with confidence intervals, effect sizes, degrees of freedom and *P* value noted<br>*Give P values as exact values whenever suitable.*
☒	☐	For Bayesian analysis, information on the choice of priors and Markov chain Monte Carlo settings
☒	☐	For hierarchical and complex designs, identification of the appropriate level for tests and full reporting of outcomes
☐	☒	Estimates of effect sizes (e.g. Cohen's *d*, Pearson's *r*), indicating how they were calculated

Our web collection on statistics for biologists contains articles on many of the points above.

Software and code

Policy information about availability of computer code

Data collection	No custom code was generated in this study.
Data analysis	For RNAseq: FASTQ files were aligned to the human genome GRCh38 (GRCh38.d1.vd1.fa) using STAR v2.4.2. Transcript abundance for each sample was quantified with Salmon v0.1.19 against the transcriptome defined by Gencode v22. Gene-level counts were aggregated across isoforms, and genes with low counts (maximum expression < 10) were filtered out for downstream analyses. Differential expression analysis was performed with DESeq2 (v4.2) in R. GSEA analysis and GO analysis were performed by using the WebGestalt (WEB-based Gene SeT AnaLysis Toolkit).<br>For ChIP-seq: ChIP-seq libraries (Illumina) were prepared according to manufacturer's manual and sequenced at CRI's Sequencing Facility at UT Southwestern using single-end 50bp reads. Reads were filtered for adapter contamination using Cutadapt (Martin, 2011) and further screened to ensure that at least 90% of bases in each read had a quality score > 20. Duplicate sequences were capped at a maximum of five occurrences, and reads were aligned to the reference genome (hg19) using STAR version 2.5.2b, retaining only primary alignments. Reads overlapping blacklisted genome regions were removed, and remaining reads were extended in silico to a fragment size of 250 bp. Regions of significant enrichment relative to input control were identified with MACS2. Motif analysis was conducted using HOMER, comparing the 200 bp surrounding each peak midpoint to the 200 bp flanking sequences on either side of each peak.<br>For CRISPR screen: raw reads were initially mapped to a reference list of all GeCKOv2 sgRNA sequences and quantified for each tissue sample. MAGeCK v0.5.4 was then used with default parameters to process the sgRNA read counts, generating gene-level rankings and p-values for enrichment analysis in lung or liver metastatic lesions relative to corresponding primary tumors. Genes essential for regulating lung or liver metastasis were identified by comparing MAGeCK results across five biological replicates; genes were selected if more than four replicates showed consistent organotropism for metastasis upon sgRNA-mediated gene ablation in UMRC2 or A498 cells. Essentiality scores for these genes were calculated as the negative logarithm of their p-values for sgRNA enrichment in the preferred metastatic site (lung vs. liver or vice versa). The top ten genes with the highest essentiality scores across both ccRCC cell lines were selected for further investigation.

For single cell migration tracking data: the ImageJ Manual Tracking Plugin was used to track cell motility and tab-delimited text files were imported into the Chemotaxis and Migration Tool 2.0 (Ibidi) for plotting and quantitative analysis of Euclidean distance, velocity, and directionality. Statistical analyses were performed using GraphPad Prism 9.0 software.
For immunofluorescence data: the average intensity was quantified using the ImageJ software (Fiji), and the average filament number and F-actin length were quantified by applying the FilamentSensor 2.0 based on Java.

For manuscripts utilizing custom algorithms or software that are central to the research but not yet described in published literature, software must be made available to editors and reviewers. We strongly encourage code deposition in a community repository (e.g. GitHub). See the Nature Portfolio guidelines for submitting code & software for further information.

Data

Policy information about availability of data

All manuscripts must include a data availability statement. This statement should provide the following information, where applicable:

- Accession codes, unique identifiers, or web links for publicly available datasets
- A description of any restrictions on data availability
- For clinical datasets or third party data, please ensure that the statement adheres to our policy

All data generated or analyzed during this study are included in Figures 1-7 and Extended Data Fig 1-10. Genomics data produced by this study, including ChIP-Seq, ATAC-seq and RNA-Seq, the raw data has been deposited in the Gene Expression Omnibus (GEO) under accession number GSE272563, link: https://www.ncbi.nlm.nih.gov/geo/query/acc.cgi?acc=GSE272563
The raw data of CRISPR screening has been deposited in NCBI under accession number PRJNA1161221, link: https://www.ncbi.nlm.nih.gov/bioproject/PRJNA1161221/ .
The clinical RNA-seq data of normal and ccRCC tumor was obtained from Dr. Payal Kapur were deposited in EGA under the accession number EGAS00001005516, link: https://ega-archive.org/studies/ .
The ChIP-seq data of H3K4me1 and H3K27ac in parental 786-O and 786-O M1A cells were obtained from GEO under the accession code GSE98015, link: https://www.ncbi.nlm.nih.gov/geo/query/acc.cgi .

Research involving human participants, their data, or biological material

Policy information about studies with human participants or human data. See also policy information about sex, gender (identity/presentation), and sexual orientation and race, ethnicity and racism.

Reporting on sex and gender	ccRCC patient tissue samples were collected from both male and female patients, with no gender-based selection bias.
Reporting on race, ethnicity, or other socially relevant groupings	All experiments were designed without bias related to race, ethnicity, or other socially relevant group classifications.
Population characteristics	N/A
Recruitment	N/A
Ethics oversight	The deidentified fresh-frozen human paired (ccRCC and adjacent normal tissue) samples used in this study were obtained from the UT Southwestern tissue management core, and have been reviewed by The UT Southwestern Human Research Protection Program (HRPP), which determined that the analysis does not meet the definition of human subject research under 45 CFR 46.102 and therefore does not require Institutional Review Boards (IRBs) approval or oversight.

Note that full information on the approval of the study protocol must also be provided in the manuscript.

Field-specific reporting

Please select the one below that is the best fit for your research. If you are not sure, read the appropriate sections before making your selection.

☒ Life sciences ☐ Behavioural & social sciences ☐ Ecological, evolutionary & environmental sciences

For a reference copy of the document with all sections, see nature.com/documents/nr-reporting-summary-flat.pdf

Life sciences study design

All studies must disclose on these points even when the disclosure is negative.

Sample size	For all experiments, we selected sample sizes sufficient to ensure reproducibility of our findings and to enable meaningful statistical analyses (minimum n=3). All sample sizes are clearly indicated in the manuscript.
Data exclusions	No data was excluded from the analysis.
Replication	All experimental findings were reproducibly observed independently, with results confirmed more than two or three times in the same system and across different models, including various cell lines.

Randomization	Animals were randomly assigned to experimental groups for relevant animal experiments. Randomization was not applicable for in vitro experiments.
Blinding	Blinding was not possible during group allocation, as investigators were responsible for assigning subjects. However, blinding was implemented during data collection and analysis.

Reporting for specific materials, systems and methods

We require information from authors about some types of materials, experimental systems and methods used in many studies. Here, indicate whether each material, system or method listed is relevant to your study. If you are not sure if a list item applies to your research, read the appropriate section before selecting a response.

Materials & experimental systems

n/a	Involved in the study	
☐	☒	Antibodies
☐	☒	Eukaryotic cell lines
☒	☐	Palaeontology and archaeology
☐	☒	Animals and other organisms
☐	☒	Clinical data
☒	☐	Dual use research of concern
☒	☐	Plants

Methods

n/a	Involved in the study	
☐	☒	ChIP-seq
☒	☐	Flow cytometry
☒	☐	MRI-based neuroimaging

Antibodies

Antibodies used	Rabbit anti-HA-Tag, Cell Signaling Technology, Cat# 3724, 1:1000 dilution for WB, 1:50 for ChIP<br>Rabbit anti-Flag-Tag, Cell Signaling Technology, Cat# 14793, 1:1000 dilution for WB<br>Rabbit anti-V5-Tag, Cell Signaling Technology, Cat# 13202, 1:1000 dilution for WB<br>Rabbit anti-LPXN, Lsbio, Cat# LS-C313296, 1:1000 dilution for WB<br>Rabbit anti-H3K4me3, Abcam, Cat# ab8580, 1:1000 dilution for WB<br>Rabbit anti-H3K9ac, Abcam, Cat# ab10812, 1:1000 dilution for WB<br>Mouse anti-Dnmt1, Santa Cruz Biotechnology, Cat# sc-271729, 1:200 dilution for WB<br>Rabbit anti-H3K27ac , Cell Signaling Technology, Cat# 4353, 1:1000 dilution for WB<br>Rabbit anti-H3, Abcam, Cat# ab1791, 1:1000 dilution for WB<br>Rabbit anti-HLF N1, Lu Wang lab, Custom Antibody, 1:1000 dilution for WB, 1:3000 dilution for IHC<br>Rabbit anti-HIF-1β/ARNT, Cell Signaling Technology, Cat# 3414, 1:1000 dilution for WB<br>Rabbit anti-SMARCA4/Brg1, Cell Signaling Technology, Cat# 49360, 1:1000 dilution for WB<br>Rabbit anti-Paxillin, Cell Signaling Technology, Cat# 2542, 1:1000 dilution for WB<br>Rabbit anti-Phospho-Paxillin (Tyr118), Cell Signaling Technology, Cat# 69363, 1:1000 dilution for WB, 1:400 dilution for IF<br>Mouse anti-α-Tubulin, Cell Signaling Technology, Cat# 3873, 1:1000 dilution for WB<br>Mouse anti-Vinculin, Sigma-Aldrich, Cat# V9131, 1:1000 dilution for WB<br>Rabbit anti-LPXN, ATLAS ANTIBODIES, HPA061441, 1:500 dilution for IF<br>HRP-conjugated goat anti-mouse, Thermo Fisher Scientific, Cat# 31430, 1:5000 dilution for WB<br>HRP-conjugated goat anti-rabbit IgG, Thermo Fisher Scientific, Cat# 31460, 1:5000 dilution for WB
Validation	Pre-validated antibodies were purchased from reputable commercial sources. For the custom rabbit anti-HLF N1 antibody, it has been first validated by WB in HepG2 cells with HLF deletion by sgRNAs; for its application in IHC, it has been validated by comparing its signal in 786-O cell blocks transfected with either an empty vector or HLF, confirming stronger staining in the HLF-overexpressing cells.

Eukaryotic cell lines

Policy information about [cell lines and Sex and Gender in Research]

Cell line source(s)	786-O, A498, 293T, A375-MA2, H1299 and Caki-1 were purchased from the American Type Culture Collection (ATCC), UMRC2 and UMRC6 were purchased from Sigma-Aldrich, HK-2 and RPTEC was acquired from Peter Ly lab at UTSW, 143B was acquired from Tao Yue lab at UTSW, HepG2 was acquired from Lu Wang lab at Northwestern University.
Authentication	Cell lines purchased from ATCC and Sigma-Aldrich were authenticated using short tandem repeat (STR) profiling. Cell lines obtained from other labs, however, were not re-authenticated upon receipt.
Mycoplasma contamination	All cell lines were tested negative for mycoplasma by using the mycoplasma detection kit (Lonza, LT07–218) or mycoplasma elimination reagent-plasmocin (Invivogen, ant-mpt).

Commonly misidentified lines (See ICLAC register)	No commonly misidentified lines were used in this paper.

Animals and other research organisms

Policy information about studies involving animals; ARRIVE guidelines recommended for reporting animal research, and Sex and Gender in Research

Laboratory animals	Male and female NOD.CB17-Prkdc(scid) Il2rg(tm1Wjl)/SzJ (NSG) mice aged six to eight weeks old were used in this study. Mice were purchased from Jackson Laboratories. Mice were housed in the ARC Mouse Facility at UT Southwestern under specific-pathogen-free (SPF) conditions, with climate control and a 12-hour light/dark cycle.
Wild animals	No wild animals were used in this study.
Reporting on sex	Both male and female mice were used for orthotopic and subcutaneous transplantation, while female mice were used for intravenous injection in this study. Data was not analyzed for sex specific differences as the goal was to assay metastasis.
Field-collected samples	No field-collected samples were used in this study.
Ethics oversight	All animal experiments were approved by and performed in accordance with the guidelines of the Institutional Animal Care and Use Committee (IACUC) from UT Southwestern Medical Center (approved protocol number: 2019-102794).

Note that full information on the approval of the study protocol must also be provided in the manuscript.

Clinical data

Policy information about clinical studies

All manuscripts should comply with the ICMJEguidelines for publication of clinical research and a completedCONSORT checklist must be included with all submissions.

Clinical trial registration	N/A
Study protocol	N/A
Data collection	N/A
Outcomes	N/A

Plants

Seed stocks	N/A
Novel plant genotypes	N/A
Authentication	N/A

ChIP-seq

Data deposition

☒ Confirm that both raw and final processed data have been deposited in a public database such as GEO.

☒ Confirm that you have deposited or provided access to graph files (e.g. BED files) for the called peaks.

Data access links *May remain private before publication.*	https://www.ncbi.nlm.nih.gov/geo/query/acc.cgi?acc=GSE272563
Files in database submission	GSM8404637 786-O Lentivirus Empty Vector RNA Rep1<br>GSM8404638 786-O Lentivirus Empty Vector RNA Rep2<br>GSM8404639 786-O Lentivirus Empty Vector RNA Rep3<br>GSM8404640 786-O Lentivirus HLF (Overexpression) RNA Rep1<br>GSM8404641 786-O Lentivirus HLF (Overexpression) RNA Rep2<br>GSM8404642 786-O Lentivirus HLF (Overexpression) RNA Rep3<br>GSM8404643 786-O sgCtrl RNA Rep1

GSM8404644 786-O sgCtrl RNA Rep2
GSM8404645 786-O sgCtrl RNA Rep3
GSM8404646 786-O sgHLF RNA Rep1
GSM8404647 786-O sgHLF RNA Rep2
GSM8404648 786-O sgHLF RNA Rep3
GSM8404649 786-O-P ATAC Rep1
GSM8404650 786-O-P ATAC Rep2
GSM8404651 786-O-LM ATAC Rep1
GSM8404652 786-O-LM ATAC Rep2
GSM8972938 786-O HA-HLF ChIP Rep1
GSM8972939 786-O HA-HLF ChIP Rep2
GSM8972940 786-O HA-HLF input Rep1
GSM8972941 786-O HA-HLF input Rep2

Genome browser session
(e.g. UCSC)

We only deposit data on GEO and not in the Genome Browser session.

Methodology

Replicates

Two biological replicates (786-O cells) were used for both the input and HA-ChIP groups.

Sequencing depth

Nextseq 2000, paired-end sequencing with 75 bp reads was used in this study.

Antibodies

Rabbit anti-HA-Tag, Monoclonal antibody, Cell Signaling Technology, Cat# 3724

Peak calling parameters

Peak calling was performed using MACS2 (macs2 callpeak -f BAM -g hs --keep-dup 1)

Data quality

FastQC

Software

samtools (v1.21), MACS(v2.1.2), deeptools (v3.2.0), bedtools (v2.30), python (v3.10.9)
